# Beneficial effects of a *Coriolus versicolor*-based vaginal gel on cervical epithelization, vaginal microbiota and vaginal health: a pilot study in asymptomatic women

**DOI:** 10.1186/s12905-017-0374-2

**Published:** 2017-03-16

**Authors:** Santiago Palacios, Fernando Losa, Damián Dexeus, Javier Cortés

**Affiliations:** 1 Gynecology and Obstetrics Department, Instituto Palacios de Medicina y Salud de la Mujer, C/Antonio Acuña 9, E-28009 Madrid, Spain; 2 Gynecology and Obstetrics Department, Clínica Sagrada Familia, Barcelona, Spain; 3 Gynecology and Obstetrics Department, SOMDEX-Clínica Tres Torres, Women´s Health Institute, Barcelona, Spain; 4 Senior Consultant on Gynecologic Oncology. Former President of the Spanish Association of Colposcopy and Cervical Pathology, Palma de Mallorca, Spain

**Keywords:** Vaginal gel, *Coriolus versicolor*, Niosomes, Cervical epithelization, Vaginal microbiota, Vaginal health

## Abstract

**Background:**

To assess the effect of a 12-day treatment using a vaginal gel based on niosomes containing hyaluronic acid, ß-glucan, alpha-glucan oligosaccharide, *Coriolus versicolor*, *Asian centella*, *Azadirachta indica* and *Aloe vera* on vaginal microbiota, cervical epithelization and vaginal health.

**Methods:**

Open-label, prospective pilot study conducted in asymptomatic women in daily practice. Cervical epithelization was evaluated by colposcopy using an ectopy epithelization score (from 5: no ectopy to 1: severe ectopy and bleeding), vaginal microbiota using the VaginaStatus-Diagnostic test (Instiüt für Mikroökologie, Herborn, Germany) and further rated by the investigator using a 5-point Liker scale (from 5: normal to 1: very severe deterioration in which all evaluated species were altered), and vaginal health using the Vaginal Health Index.

**Results:**

In 21 women, a positive effect to improve epithelization of the cervical mucosa, with a mean score of 4.42 at the final visit as compared to 3.09 at baseline (*P* < 0.0001) (43% improvement). In 10 women, there was a trend of improving of vaginal microbiota status, with a mean score of 4.0 at the final visit vs. 3.3 at baseline (*P* = NS) (21.2% improvement). In 11 women, the Vaginal Health Index increased from 19.0 at baseline to 22.3 at the final visit (*P* = 0.007). The concentration of *Lactobacillus* spp. increased 54.5% of women and pH decreased from 4.32 to 4.09.

**Conclusions:**

These encouraging preliminary results provide the basis for designing a randomized controlled study, and for potential use in human papilloma virus infection.

**Trial registration:**

ISRCTN77955077. Registration date: February 15, 2017. Retrospectively registered

## Background

The human female genital tract which includes the vaginal fluids present in the cervico-vaginal mucosa contain all essential elements for a number of functions, such as response against genital pathogens, re-epithelization of lesions and maintenance of general vaginal health. The vaginal ecosystem plays an important role in preventing urogenital infections, and alterations of this dynamic environment causes vaginal dysbacteriosis [1, 2]. A healthy vaginal ecosystem depends on a normal microbiota mainly consisting of *Lactobacillus* spp., a sufficient estrogen-dependent maturation of the vaginal epithelium and an intact local immunity [3]. The vaginal microbiota environment significantly affects the host’s general state of health by acting as a biological barrier to infectious agents [4]. Preservation of other functions, such as hydration conditions of vaginal tissues, pH, temperature, and characteristics of the cervical mucus have been also recognized to be important for the local activities of the innate and cell-mediated immune systems of vaginal epithelial cells [5, 6].

To maintain a healthy vaginal ecosystem or to restore any disturbance, sufficient estrogen levels, an intact mature vaginal epithelium, and physiological *Lactobacillus* microbiota have been reported to be essential. It has been shown that topical combinations of lactobacilli and estrogens can be safely used in pre- and postmenopausal women for the restoration of the vaginal microbiota after anti-infective therapy, for treatment of symptomatic vaginal atrophy, and for abnormal vaginal flora therapy [7, 8]. Local estrogen treatment restores the cytology pH and vascularity of the vagina, resulting in resolution of symptoms of vaginal atrophy [9, 10]. Topical estrogen therapy for the treatment of atrophic vaginitis in postmenopausal women has been recommended by scientific societies and expert panel consensus [11, 12].

The concept of ‘vaginal health’ as the vaginal state in which the physiological conditions and the vaginal microenvironment and microbiota are not disrupted, has gained increasing interest in recent years and is also being extended to healthy women. Traditionally, natural products have been used for vaginal health, including probiotics, essential oils, propolis, lipid lubricans, pectins, medicinal plant extracts with phytoestroestrogenic activities and a variety of other extracts with primarily moisturizing effects [13–18].

Recently, a non-hormonal gel that acts as a moisturizer and lubricant because of strong hydrating properties, also enhancing and accelerating repair of atrophic or injured cervicovaginal mucosa has been approved in Spain as a medical device. The product is based on niosomes containing hyaluronic acid, ß-glucan, alpha-glucan oligosaccharide, *Coriolus versicolor*, *Asian centella*, *Azadirachta indica* (also known as Neem) and *Aloe vera*. Encapsulation in niosomes allows a more penetrating capability of the components, which have shown hydrating, repairing, anti-inflammatory and immunomodulatory effects as well as preserving the balance of vaginal microbiota [19–24].

A pilot study was designed to assess the clinical benefits of this *Coriolus versicolor*-based vaginal gel on epithelization of cervical lesions and to improve vaginal microbiota and vaginal health in asymptomatic healthy women.

## Methods

### Design

An open-label, non-comparative, prospective and pilot clinical study was conducted in two gynecology clinics from Madrid and Barcelona (Spain) in daily practice conditions. The objective of the study was to assess the effect of a *Coriolus versicolor*-based vaginal gel on the following parameters: a) epithelization of cervical lesions (reversion of cervical ectropion), b) composition of vaginal microbiota, and c) vaginal health. The studies were performed in accordance with the principles of the Declaration of Helsinki for the protection of human subjects, and oral informed consent was obtained from all participants. According to Spain regulations, ethics approval is not required for studies with the following characteristics: real life, pilot study, with a medical device (not a drug) class I already marketed and used within approved indication, and sponsored by the principal investigator and not by a private pharmaceutical company.

### Participants

Women aged between 18 and 45 years old attending a routine gynecological visit, without signs and symptoms of vaginal disease and a normal Papanicolaou smear were included in the study. An epithelization score between 4 and 1 was also an inclusion criterion. Exclusion criteria included vaginal infections, use of vaginal products other than the investigational compound and being pregnant or breastfeeding. Women with history or concomitant diseases who were deemed to be ineligible by the investigator were also excluded from the study.

### Study procedures

Women who agreed and met the inclusion criteria were instructed on the correct use of the vaginal gel (Palomacare®, Procare Health, Castelldefels, Barcelona, Spain) according to the manufacturer’s information leaflet supplied with the gel. In addition to the extract from *Coriolus versicolor* as the main agent, the gel contains nisosomes of hyaluronic acid (a moisturing agent), nisosomes de beta-glucan (an anti-inflammatory agent), Bioecolia® (a prebiotic agent), *Centella asiatica* (fitosomes®) (a tissue regenerating agent), *Azadirachta indica* extract (Neem) (a re-epitelizing agent), and *Aloe vera*. It was recommended to use the medical device between periods, once a day and at bedtime for 12 consecutive days. There were no restrictions regarding sexual activity including the use of condoms. The use of douches or vaginal deodorants was not allowed. The vaginal gel was supplied to all women without cost for the total duration of treatment. Participants were visited at baseline (visit 1, inclusion in the study) and after 12 days of treatment (visit 2). In both visits the degree of epithelization, composition of vaginal microbiota, *Lactobacillus* spp. concentration, vaginal pH, and the Vaginal Health Index were evaluated.

### Assessments

The degree of epithelization of the cervical mucosa was evaluated by standard colposcopy and rated by the investigator using an ectopy epithelization score, where 5 was no ectopy, 4: mild (<25% of the external os), 3: moderate (25–50% of the external os), 2: severe (>50% of the external os) and 1: severe ectopy and bleeding.

Vaginal microbiota was evaluated using the VaginaStatus-Diagnostic test (Instiüt für Mikroökologie, Herborn, Germany) [25] and further rated by the investigator using a 5-point Liker scale, in which 1 was very severe deterioration of vaginal microbiota (all evaluated species were altered); 2: severe deterioration (detection of *Candida* spp. or *Mycoplasma* spp. or alterations in more than three species but not of all species); 3: moderate deterioration (alteration of three species but no *Candida* spp., *Mycoplasma* spp., *Atopobium vaginae*, *Echerichia coli* and *Gardnerella vaginalis* could be present); 4: mild deterioration (alteration of one or two species but no *Candida* spp., *Mycoplasma* spp., *Atopobium vaginae*, *Echerichia coli* and *Gardnerella vaginalis* could be present); and 5: normal. *Lactobacillus* spp. concentrations and pH were also measured.

The Vaginal Health Index was also assessed [26]. The Vaginal Health Index is a system used to evaluate vaginal elasticity, fluid volume, pH, epithelial integrity, and moisture on a scale of 1 to 5 (Table 1). Both Vaginal Health Index and vaginal microbiota were only assessed in 11 patients included in the Barcelona investigation site for logistic and financial reasons.

**Table 1:** Vaginal health index

	1	2	3	4	5
Elasticity	None	Poor	Fair	Good	Excellent
Fluid volume (pooling of secretions)	None	Scant amount, vault not entirely covered	Superficial amount, vault entirely covered	Moderate amount of dryness (small areas of dryness on cotton-tip applicator)	Normal amount (fully saturates on cotton-tip applicator)
pH	≥6.1	5.6–6.0	5.1–5.5	4.7–5.0	<4.6
Epithelial integrity	Petechiae noted before contact	Bleeds with light contact	Bleeds with scraping	Not friable, thin epithelium	Normal
Moisture (coating)	None, surface inflamed	None, surface not inflamed	Minimal	Moderate	Normal

### Statistical analysis

Because of the exploratory characteristics of the study, sample size calculation was not mandatory. Categorical variables are expressed as numbers and percentages, and quantitative variables as mean and standard deviation (SD). The Wilcoxon signed-rank test was used for the comparison of paired samples of continuous data. Data were analyzed with the Power Analysis and Sample Size software program, version 2011.

## Results

### Epithelization of the cervix

**Fig. 1:**
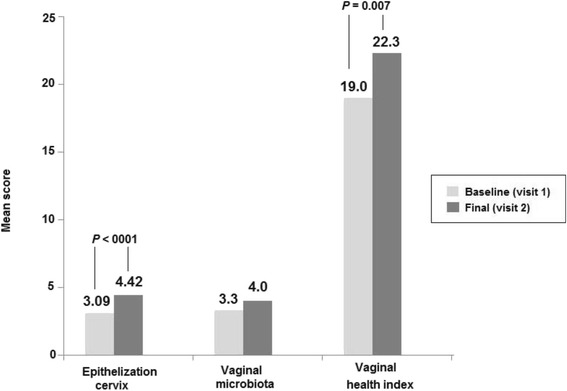
Changes in the study parameters before and after 12-day treatment with a Coriolus versicolor-based vaginal gel

**Fig. 2:**
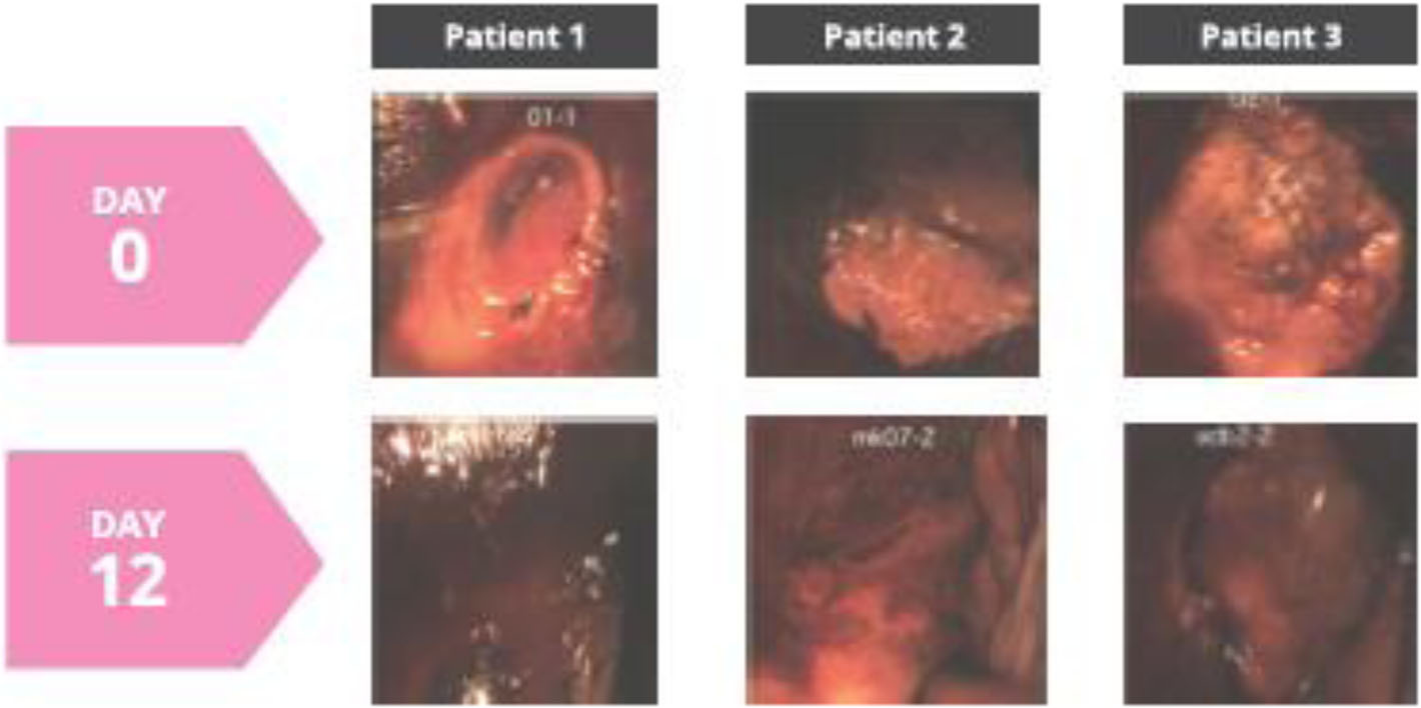
Colposcopic images of three patients before and after applying test product

A total of 21 women with a mean age of 32.6 years (range 20–43 years) participated in evaluation of epithelization of the ectocervix. Treatment with the *Coriolus versicolor*-based vaginal gel showed a positive effect to improve reepithelization of the cervical mucosa, with a mean score of 4.42 at the final visit as compared to 3.09 at baseline (*P* < 0.0001), and an overall improvement of 43% (Fig. 1). Also, at the end of the study, 95.3% of women showed an improvement in the epithelization degree, with a score of 5 observed in 11 women (52.4%) (Table 2). Changes in colposcopic images before and after treatment are shown in Fig. 2.

**Table 2:** Results of colposcopy score

Patients	Colposcopy epithelization score^a^	
	Baseline (visit 1)	End of study (visit 2)
1	3	5
2	3	4
3	3	4
4	1	3
5	2	5
6	3	3
7	3	4
8	2	4
9	4	5
10	3	4
11	4	5
12	4	5
13	4	5
14	3	5
15	5	5
16	4	5
17	2	4
18	4	5
19	3	5
20	2	4
21	3	4

^a^5-point Likert scale from 5: no ectopy to 1: severe ectopy with bleeding

### Vaginal microbiota

Out of the 11 patients recruited, ten women aged between 20 and 43 years (mean age 32.8 years) were included in the assessment of vaginal microbiota (one exclusion due to sample manipulation error). At the baseline visit, 9 of 10 patients (90%) showed some alterations in the vaginal microbiota (*Gardnerella vaginalis*, *E. coli*, *Streptococcus* spp. etc.) despite of being clinically asymptomatic. There was trend of improving of vaginal microbiota status, with a mean score of 4.0 at the final visit vs. 3.3 at baseline (*P* = NS), and an improvement of 21.2% (Fig. 1). At the end of the study, 5 patients improved their vaginal status score with 4 of them reaching a 5-point score (normal status). One patient worsened and the remaining 4 showed no changes (Table 3).

**Table 3:** Results of vaginal microbiota

Patients	Vaginal microbiota score^a^	
	Baseline (visit 1)	End of study (visit 2)
1	4	5
2	3	3
3	2	5
4	4	5
5	5	5
6	2	4
7	3	3
8	4	2
9	3	3
10	3	5

^a^5-point Likert scale from 5: no deterioration of vaginal microbiota (normal) to 1: very severe deterioration (alteration of all species evaluated)

### Vaginal health

A total of 11 women aged between 25 and 43 years (mean age 32.6 years) were included in the assessment of vaginal health. There was a significant increase in the Vaginal Health Index from a mean of 19.0 at baseline to 22.3 at the final visit after 12 days of treatment (*P* = 0.007) (Fig. 1). Also the concentration of *Lactobacillus* spp. increased in 6 patients (54.5%) and no changes were observed in 1. In the remaining 3 women, a decrease in *Lactobacillus* concentration was found. The mean vaginal pH showed a trend to decrease (4.32 vs. 4.09, *P* = NS) (Table 4).

**Table 4:** Changes in *Lactobacillus* spp. concentration and pH

Patients	*Lactobacillus* spp.		Vaginal pH	
	Baseline (visit 1)	End of study (visit 2)	Baseline (visit 1)	End of study (visit 2)
1	1 × 10^8^	2 × 10^8^	4.2	5.4
2	3 × 10^8^	5 × 10^8^	4.8	4.2
3	2 × 10^9^	3 × 10^9^	3.9	3.6
4	5 × 10^8^	5 × 10^8^	4	3.8
5	5 × 10^9^	5 × 10^9^	3.8	3.6
6	<5 × 10^9^	3 × 10^8^	5.2	3.8
7	5 × 10^8^	<5 × 10^5^	4.7	4.4
9	1 × 10^8^	3 × 10^8^	4	4.2
10	<5 × 10^5^	5 × 10^5^	4.2	4
11	4 × 10^8^	3 × 10^8^	4	4

Patient #8 was excluded because of sampling handling error

Adverse effects due to using the vaginal gel were not recorded.

## Discussion

The present findings showing a clear beneficial effect of the *Coriolus versicolor*-based vaginal gel on epithelization of the ectocervix, vaginal microbiota and vaginal health, are highly encouraging for the design of a prospective randomized controlled study in order to confirm the results reported. If the multi-protective action of the vaginal gel observed in this pilot experience would be confirmed in a further randomized trial, clinical relevant effects in the mechanisms of entry and persistence of human papilloma virus (HPV) in the mucosal epithelium [27, 28] might be documented. Scientific evidence accumulated from virological, molecular, clinical and epidemiological studies has identified HPV as the primary etiological agent in cervical cancer [29–31].

In fact, the protection against viral aggression is based on three actions of the vaginal gel here described. Firstly, normalization of the vaginal microbiota. It is well known that bacterial vaginosis linked to an imbalance of vaginal ecosystem predisposes women to infection by HPV [32]. Palomacare® presents promising evidence of the correcting and stabilizing effects on vaginal microbiota. Secondly, the immunomodulatory properties. Polysaccharides and ß-glucans from *Coriolus versicolor*, one of the components of the vaginal gel, have been shown to have antioxidant, immunomodulatory and anti-tumor properties [33, 34]. Moreover, extracts of *Coriolus versicolor* appear to be a lymphocyte mitogen by differentially enhancing the production of Th1-related cytokines [35]. A Th1 response has been shown to be important for clearance of HPV infection [36]. Thirdly, improvement of epithelization at the squamocolumnar junction in the ectocervix with subsequent reduction of mitotic activity may also decrease cell susceptibility to HPV infection [37].

The present results should be interpreted taking into account the pilot nature of the study, the absence of a control group and the short-term use of the product. Also, a comparison with previous studies is not presented as we report preliminary results of a pilot study with a new vaginal gel. However, these encouraging preliminary findings provide the basis for designing a randomized controlled study, and subsequently in the presence of confirmatory data, for potential use in HPV taking the advantages of easy and comfortable administration of the product.

## Conclusions

In a pilot study conducted among asymptomatic women, the use of a vaginal gel based on *Coriolus versicolor* and other ingredients such as niosomes containing hyaluronic acid, ß-glucan, alpha-glucan oligosaccharide, *Asian centella, Azadirachta indica* and *Aloe vera* for 12 consecutive days showed positive effects toward improving vaginal microbiota, cervical epithelization and vaginal health. Further studies must be conducted to confirm these positive results, as well as to evaluate the potential use in human papilloma virus infection. Moreover, comparative studies with vaginal gels composed of estrogen and topical microbicides would be of interest.

## Abbreviation

HPV: Human papilloma virus
